# ‘More evolved than you’: Evolutionary spirituality as a cultural frame for psychedelic experiences

**DOI:** 10.3389/fpsyg.2023.1103847

**Published:** 2023-03-27

**Authors:** Jules Evans

**Affiliations:** Centre for the History of the Emotions, Queen Mary University of London, London, United Kingdom

**Keywords:** psychedelics, psychedelic history, eugenics, transhumanism, spiritual eugenics, evolutionary spirituality, New Age spirituality

## Abstract

One of the dominant cultural frames for psychedelics in western culture over last 130 years has been evolutionary spirituality. This tradition suggests human evolution is not finished and can be guided towards the creation of higher beings through such techniques as psychedelics and eugenics or genetic modification. But is everyone evolving into a new species, or just an elite? This essay defines the tradition of evolutionary spirituality and points to five of the ethical limitations of the tradition – its tendency to spiritual narcissism, contempt for the less-evolved masses, Social Darwinism and Malthusianism, spiritual eugenics, and illiberal utopian politics—before suggesting responses to these limitations.

## 1. Defining evolutionary spirituality

One of the dominant cultural frames for psychedelics in western culture over the last 130 years has been evolutionary spirituality (Elcock, 2013). This is a tradition within the broader culture of New Age spirituality, which seeks to synthesize spirituality with evolutionary theory, and which asserts that human evolution is not finished and can be guided towards the creation of higher beings through such techniques as meditation, psychedelics and eugenics or genetic modification (Ferguson, 1981, pp. 157–162; Herrick, 2003, pp. 118–148; Huston, 2007; Hubbard, 2015).

Evolutionary spirituality has historical roots in eighteenth-century ideas of the improvement and possible perfectibility of humans. Scottish enlightenment philosophers such as Adam Smith and Adam Ferguson suggested cultures naturally evolve into higher stages, from hunter-gathering to farming to industrial capitalism (Palmeri, 2013, pp. 1–26). William Godwin and the Marquis de Condorcet predicted science could advance so far that humans would become immortal, blissful beings (Chonaill, 2007). The Reverend Thomas Malthus, while criticizing Godwin’s utopianism, put forward his own version of evolutionary progressivism, writing that the world is a “mighty process for the creation and formation of mind’ in which malformed specimens get broken while ‘those vessels whose forms are full of truth, grace and loveliness, will be wafted into happier situations, near the presence of the mighty maker” (Malthus, 1966, p. 247). In the 19^th^ century, German idealists like Fichte, Schelling and Hegel thought that God or Spirit evolved in a dynamic process throughout human history, manifesting in higher forms in particular individuals and cultures (Murphy, 2013). This idea was taken up by transcendentalists in other countries, like Ralph Waldo Emerson, who admired “the German thought of the Progressive God, who has got thus far with his experiment, but will get out yet a triumphant and faultless race” (Emerson, 1975, p. 263).

Charles Darwin’s theory of evolution *via* natural selection was a challenge to these conceptions of a progressive moral evolution (Bowler, 2000, p. 524–532). Evolution in Darwin’s theory is an automatic process without any values or moral goals, in which most species go extinct, and only those who are suited to changing circumstances survive. There is no such thing as a ‘higher’ species, and ‘fitter’ simply means ‘able to survive and reproduce under particular circumstances’. Nonetheless, the triumph of Darwinism inspired countless more progressivist and spiritual versions of evolution to arise, ‘substitute-religions’ (Rose, 1986, pp. 1–4), which tried to find new sources of meaning, myth, ethics and purpose in an evolutionary universe (Bowler, 2003, pp. 237; Midgley, 1985, pp. 15–19). These science-religions appealed to the authority of evolution as previous religions had appealed to the authority of God.

One could write a large volume on the varieties of evolutionary spirituality that have emerged in the last 160 years since the publication of *The Origin of Species:* Herbert Spencer’s ‘religion of the Unknowable’, Alfred Russell Wallace’s evolutionary Spiritualism, Friedrich Nietzsche’s cult of the *ubermensch,* Ernst Haeckel’s Monism, Henri Bergson’s creative evolution, the evolutionary occultism of the Hermetic Order of the Golden Dawn, the holism of Jan Smuts, the mystical-evolutionary psychology of Frederic Myers and William James, the process philosophy of Alfred North Whitehead, Sri Aurobindo’s Integral Yoga, Helena Blavatsky’s Theosophy, Rudolf Steiner’s Anthroposophy, HG Wells’ Utopianism, Julian Huxley’s evolutionary humanism (later called transhumanism) and Teilhard de Chardin’s Christian evolutionary mysticism are all examples of pre-war evolutionary spiritualities.

After World War Two, one could point to the various forms of the human potential movement (as articulated by Abraham Maslow, Aldous Huxley, Michael Murphy and others), the transpersonal psychology of Stanislav Grof and others, Ken Wilber’s Integral Theory, the evolutionary spirituality put forward in the 1990s by figures like Andrew Cohen and Barbara Marx Hubbard; some forms of Deep Ecology and ‘Gaia-religion’^1^, and finally the different variants of transhumanism which emerged in the 1970s-1990s and have become popular with the Silicon Valley elite today. Within this roll-call of evolutionary spiritualities, one should include eugenics, the ‘religion of the future’ as many of its apostles called it. Its leading prophet, Francis Galton, described the ‘creed’ of eugenics as the first post-Darwinian evolutionary religion (Galton, 1883, p. 304).

These variants of evolutionary spirituality share two ideas. First, it’s possible and desirable to combine science and religion into a new synthesis. This, it is believed, could eventually replace Christianity and other traditional religions and become the global religion of the future. Second, human evolution is an ongoing process, which can be guided to higher and better forms. Apostles of evolutionary spirituality think humans have the potential to evolve into superbeings, called things like the New Man, the *ubermensch*, *homo deus*, ‘the coming race’, the future human, the transhuman, the Self-Actualized Person, or perhaps a collective stage of consciousness, such as the Noosphere, Super-Intelligence, the Super-Mind or the Singularity.

Most proponents of evolutionary spirituality are non-materialist in their metaphysics, although not all are. But on the whole, they see evolution as a spiritual force, as divinity unfolding in matter, or matter evolving into gods. They do not all think humans will inevitably evolve into superhumans – for some (like HG Wells) it’s an open-ended question if *homo sapiens* will evolve or degenerate. They also have different ideas of how evolution takes place and can be steered. Most believe in natural selection, but some also support artificial selection (eugenics or genetic modification). And many champions of evolutionary spirituality believe in psychological, cultural and spiritual evolution, which supposedly takes place not through heredity but through ideas, books, spiritual practices and ecstatic experiences.

Proponents of evolutionary spirituality generally embrace a variant of the theory of evolution first put forward by Jean-Baptiste Lamarck in his *Philosophie zoologique* of 1809 (Lamarck, 2011) in which physical characteristics acquired in your lifetime can be passed on to your descendants (in the famous example, children of a blacksmith would supposedly inherit the bulging muscles he developed in his life). Lamarckian ideas were and are quite common, including among leading scientists – Darwin himself sometimes believed in the heredity of acquired characteristics (Bowler, 2000, p. 525). Champions of evolutionary spirituality expand Lamarckism to include mental and spiritual traits, so that the attainment of a ‘higher state of consciousness’ can mark an advance in evolution and even the emergence of a new species (Singleton, 2007). Hence psychedelics could be one technique by which humans expand their potential and advance their evolution – an idea put forward by Albert Hofmann, Aldous and Julian Huxley, Humphrey Osmond, Timothy Leary, Ralph Metzner, Robert Anton Wilson, Terence McKenna, Stan Grof, Rick Doblin and other leading psychedelic thinkers.^2^

A good example of this way of thinking is Richard M. Bucke’s 1901 book, *Cosmic Consciousness: A Study in the Evolution of the Human Mind.* Bucke was a 19^th^-century Canadian psychiatrist, who became a devoted admirer of the poet Walt Whitman. After an evening spent reading Whitman’s poetry, Bucke had an ecstatic experience, which he described as ‘cosmic consciousness’. He felt he was born again, into the higher species to which Whitman belonged. Bucke then traced occurrences of cosmic consciousness in religious literature and contemporary reports, and came to the conclusion these experiences were becoming more common, especially among his circle. He concluded that a new species was emerging, as superior to *homo sapiens* as humans are to dogs, and this new species would eventually take over the world. Some humans would join the new species, while other individuals and races would not (Bucke, 2000, p. 64). This idea of being born again through a spiritual experience perhaps owes more to ecstatic Christianity and Gnosticism than Darwinism, but nonetheless, Bucke presented his religious worldview as an evidence-based evolutionary hypothesis, and it influenced subsequent scientists like William James, Abraham Maslow and Timothy Leary.

Another important difference between evolutionary spirituality and Darwinism is that, rather than believing in the Darwinian conception of evolution as a branching tree leading in multiple directions, believers in evolutionary spirituality are more likely to embrace the pre-Darwinian idea of the ‘great chain of being’, which has historical roots in Plato, Aristotle, Plotinus and Pseudo-Dionysus (Lovejoy, 1936; Wilber, 1993; Murphy, 2013). According to this theory, there is a natural-spiritual hierarchy from plants to animals to humans to angels and finally to God. Some humans are higher up the hierarchy than others. Through spiritual practice, we can ascend the evolutionary escalator, realize new ‘potentialities’, and perhaps become god-like.

## 2. Five ethical issues with evolutionary spirituality

There is much to admire in evolutionary spirituality. It attempts to resolve the conflict between science and religion, and to combine science and spirituality into a new synthesis. It finds a way to re-connect human values with nature and the cosmos, giving its followers a sense of meaning and hope for humanity’s long-term future. It has an optimistic sense of humanity’s potential to evolve into god-like beings. The tradition often overlaps with optimistic and progressive political attitudes: humans can solve war, disease, environmental degradation and death, and build a planetary civilization in harmony with nature, before perhaps exploring the universe. Its supporters have, over the last 160 years, arguably been less prone to misogyny and homophobia than older world religions (although, as we’ll see, still prone to racial and class prejudice). Nonetheless, there are some potential ethical issues with this cultural frame. This essay will address five: an inclination to spiritual narcissism, a contempt for those seen as less evolved, a tendency to social Darwinism and Malthusianism, a tendency to ‘spiritual eugenics’, and finally a propensity for illiberal utopian politics. I will illustrate these points with examples from leading figures in the tradition, before suggesting exceptions and responses.

### 2.1. Spiritual narcissism

Followers of evolutionary spirituality believe that all beings exist in a bell-curve of self-actualization. Some humans are more evolved, more conscious, more vital, more ‘fully human’ (Maslow, 1971, p. 45). This often leads to the idea of an evolutionary elite, what Abraham Maslow called “advance scouts for the race” (Ferguson, 1981, p. 56). The concept of a hierarchy of initiation leading to a spiritual elite is found in many religions, of course. And obviously, other religions’ idea of ‘the elect’ can lead to spiritual narcissism and casteism. However, it is my hypothesis that evolutionary spirituality leads to *higher* collective spiritual narcissism than other religions, due to two beliefs.

Firstly, following Friedrich Nietzsche (who has exerted a huge influence on the tradition), apostles of evolutionary spirituality tend to reject humility and self-abasement as important virtues, and instead celebrate humanity’s capacity to become gods (Dunnington, 2019, p. 105). That’s not necessarily a problem if one believes that all humans share this potential. But in practice, believers in evolutionary spirituality often believe only a few humans are evolving to a higher stage – especially themselves – while others are failing to evolve. Secondly, followers of evolutionary spirituality believe they are superior to the masses not because they hold certain beliefs or follow a particular lifestyle, like Christians or Muslims. They believe they are *essentially* superior, the next step in evolution, the first buds of *homo deus*. In Richard Bucke’s case, he thinks he is as superior to *homo sapiens* as humans are to dogs.

Let us take one example of this tendency to spiritual narcissism in evolutionary spirituality: Ken Wilber’s Integral Theory. Wilber’s intellectual achievements are impressive. But his theory does seem to appeal to a narcissistic strain in his followers. This is the promotion for his online course, Superhuman OS:

A small percentage of the human population, around 5%, is now undergoing a ‘quantum leap’ to this emerging stage of evolution. These rare individuals, from every corner of the globe, are now blazing a new evolutionary trail for all of us, and breaking through to new levels of consciousness and capabilities, beyond anything that human beings have ever experienced before (Wilber, 2017).

The implication is that, by buying into Wilber’s theory, you prove yourself a member of this exclusive group. Numerous former disciples have testified to a tendency for Wilberites to become ‘puffed up’ in the words of former Integral acolyte Jamie Wheal (Evans, 2021a,b,c). The author Mark Manson records his disappointment at attending an Integral conference and discovering it was a self-congratulatory talking-shop, “We’re ‘second-tier’ thinkers. We’re going to change the world … as soon as we are done talking about how awesome and ‘second-tier’ we are” (Manson, 2018). The film-maker Nora Bateson tells me she saw a similar spiritual narcissism emerge in the human potential movement:

In the 1980s, some people tried to systematize and commodify the human potential movement, to measure and quantify how realized you were and what stage of evolution you'd reached. Were you a level 5 or a level 6, orange or indigo? What level was the person you were talking to? It led to an excessive focus on the self.

Evolutionary spirituality’s tendency to collective narcissism overlaps with class privilege. Pre-war spiritual movements like the Theosophical Society, the Golden Dawn or the Society for Psychical Research tended to attract upper and middle-class affluent, educated followers (Urban, 1997), readily inclined to see themselves as more evolved than the urban proletariat. One sees a similar overlapping of spiritual narcissism and class privilege in the post-war human potential movement. Abraham Maslow, the pioneer of humanistic psychology, believed only a few humans reach the top of his hierarchy of human needs and become ‘fully human’. He found these higher beings particularly in the executive class. He worked as an in-house psychologist at one Bay Area company, Saga Corporation, where he congratulated the executives on their superior evolutionary level:

It has been suggested that only about 5% of the general population are active agents. They are the ones who run themselves and the world. It is very clear to me that every single member of this group are one of those active agents (Maslow, 1996a).

In transhumanism, a modern variant of evolutionary spirituality popular in Silicon Valley today, the business elite are sometimes as spiritually and genetically superior, almost a different species. Eliezer Yudkowsky, founder of the Rationalist movement and a leading transhumanist, recalls spending time at one venture capital conference:

these people of the Power Elite were visibly much smarter than average mortals … these CEOs and CTOs and hedge-fund traders, these folk of the mid-level power elite, seemed happier and more alive (Yudkowsky, 2008).

When the Silicon Valley business elite take psychedelics within the frame of evolutionary spirituality, it can be easy to see oneself as a highly-evolved superbeing - ‘the hallucinogenic elite’, in Eric Weinstein’s phrase (Ferriss, 2016), or what Sean Parker (the investor and psychedelic philanthropist) calls ‘immortal overlords’ (Perrigo, 2017). Christian Angermayer, the world’s leading investor in psychedelics, lives in a London penthouse apartment filled with statues of gods, emperors and immortal heroes, and aspires to divinity as well. He told Steven Bartlett’s podcast, ‘Maybe we are *meant* to play God … Maybe we are there to escape the evolutionary velocity, to be gods in our own way” (The Diary of a CEO, 2021) However, Angermayer thinks not all humans will necessarily make this leap, and there could be a bifurcation into two species—the gods and the left-behind (The Rubin Report, 2021).

Today, spiritual-but-not-religious Americans tend to be better educated than the average, with 71% of SBNRs having attended college, compared to 59% of other Americans (Lipka and Gecewicz, 2017). This educational gap could strengthen the cultural tendency to collective spiritual narcissism. Taking psychedelics at luxury retreats costing thousands of dollars, affluent psychonauts could be inclined to believe that, in the words of psychedelic author James Oroc:

We are the sharpened spearhead of humanity, we are the ones who have had what the psychologist Abraham Maslow describes as the ‘absolute peak experience’, which he believed was the ultimate achievement of being human, and something that occurs only for a tiny fraction of the human population. We are the 5% who have to help humanity move into its next phase, the recognition of our own divine origins. (Oroc, 2018, p. 125)

### 2.2. Contempt for the less-evolved or ‘unfit’ masses

This sense of evolutionary superiority is often accompanied by a tendency to look down on the masses as less evolved, less conscious, degenerate, unreal, bestial, not fully human. Again, this habit of thinking can be traced back to Friedrich Nietzsche. He suggested there is a sharp dichotomy between the ‘natural aristocracy’ and the mediocre masses. He wrote:

To me, the masses seem to be worth a glance only in three respects: first as blurred copies of great men, presented on bad paper with worn out printing plates, then as the resistance against the great men, and finally as working implements of the great. For the rest, let the devil and statistics carry them off! (Nietzsche, 1997, p. 113).

As John Carey explored, this Nietzschean contempt for the degenerate masses became fashionable among Modernist spiritual seekers like DH Lawrence. He believed in a hierarchy of self-actualization in nature, with natural aristocrats like him at the peak, and the ignorant masses below. In his novel *Kangaroo,* Lawrence writes, “The mass of mankind is soulless … Most people are dead, and scurrying and talking in the sleep of death” (Carey, 1992, p. 11). Aldous and Julian Huxley both also believed in a natural hierarchy leading to a genetic aristocracy, including the Huxleys and other talented families, with the unevolved and ignorant masses far below. Aldous Huxley wrote:

About 99.5% of the entire population of the planet are as stupid and philistine (tho’ in different ways) as the great masses of the English. The important thing, it seems to me, is not to attack the 99.5% - except for exercise – but to try to see that the 0.5% survives, keeps its quality up to the highest possible level and, if possible, dominates the rest (Huxley, 1994, p. xx).

The early transhumanist thinker and novelist HG Wells had a similar sense of a dichotomy between the intelligent minority and the idiot masses. His one-time mistress, Margaret Langer, wrote:

I am glad to agree with HG Wells when he says that the whole world at present is swarming with cramped, dreary, meaningless lives, lives which amount to nothing and which use up the resources and surplus energies of the world. (Bashford, 2014, p. 234)

Spiritual movements of the late-19^th^ and early-20^th^ century often shared this view of a spiritual-biological hierarchy in nature, with an evolved elite and the slavish masses far below. This was a view held by many members of the Hermetic Order of the Golden Dawn, for example. Israel Regardie, a disciple of Aleister Crowley who wrote a book on the Golden Dawn, declared:

The Golden Dawn is an elitist system … It is for those few who are willing to take evolution into their own hands, and make these attempts to transform themselves. The great mass of people are quite willing to drift along. They want no part or have no idea of voluntary forms of evolution (Hyatt, 1984, p. 29).

In some variants of evolutionary spirituality (certainly not all of them^3^) the bell-curve of self-actualization is racial. This is the case with Theosophy. Although the Theosophical Society aimed to be a multi-racial brotherhood of man, Madame Blavatsky told her disciples that spiritual evolution ascends through various races. Some races — particularly the Aryan race — are more evolved, soulful, ‘elect’ and ‘God-informed’, while other races in the past and today are less evolved, ‘unholy’, ‘inferior’, ‘savage’, ‘soulless’, ‘monsters’, ‘accursed’, ‘black with sin’, materialistic (Jews), more bestial or ape-like,‘degenerate in spirituality’ and possibly even demonic (Blavatsky, 2012, pp. 185, 1,157, 1,479, 1,509, 1,549, 1,551, 2,411). German Theosophists like Rudolf Steiner and Jörg Lanz von Liebenfels adapted this spiritual-evolutionary racism and pronounced the German race the most spiritually-evolved, while other races were lower on the evolutionary scale.^4^ Steiner wrote:

On one side we find the black race, which is earthly at most. We also have the yellow race, which is in the middle between earth and the cosmos. If it moves to the East, it becomes brown, attaches itself too much to the cosmos, and becomes extinct. The white race is the future, the race that is spiritually creative (Steiner, 1993, p. 67).

One can find a contempt for the less-evolved masses in later forms of evolutionary spirituality as well. Abraham Maslow, whose theory of self-actualization shows the influence of Nietzsche (Valiunas, 2011), wrote, “Only a small proportion of the human population gets to the point of identity, or of selfhood, full humanness, self-actualization” (Maslow, 1971, p. 24). Beneath that tiny percentage (which included him obviously), “it is perfectly true that the mass of society is still like a dead weight” (Maslow, 1971, p. 223).

Theories of evolutionary spirituality became popular in the psychedelic counterculture of the 1960s, thanks to figures like Timothy Leary, who suggested LSD was producing a ‘new race of mutants’ (The Harvard Crimson, 1966). This sense of oneself as a higher species could lead to psychedelic snobbery. Tom Wolfe, in *The Electric Kool-Aid Acid Test,* noted:

The world was simply and sheerly divided into ‘the aware’, those who had had the experience of being vessels of the divine, and a great mass of ‘the unaware’, ‘the unmusical,’ ‘the unattuned’ … Consciously, the Aware were never snobbish toward the Unaware, but in fact most of that great jellyfish blob of straight souls looked like hopeless cases (Wolfe, 1968, p. 131).

Wolfe overheard one psychonaut lecture a policeman at an acid test: ‘Listen, I’ve got more Awareness, more … Awareness, in my little fingernail.. My Awareness is so superior to yours that… uh…**’** (Wolfe, 1968, p. 283).

In the 1970s, Leary suggested that he and his friends were part of a ‘genetic elite’ in California (Evans, 2022b), while the rest of the world was far lower on the evolutionary scale:

To live in the East is to fail a genetic intelligence test … The folks of the Old World inhabit pre-civilized, barbarian gene-pools. Europeans and Africans and Asians are our own animal origins still obsessed with territorial conflict … The Africans are thus 2 million years behind California (Leary, 1979, p. 132).

**Figure 1:**
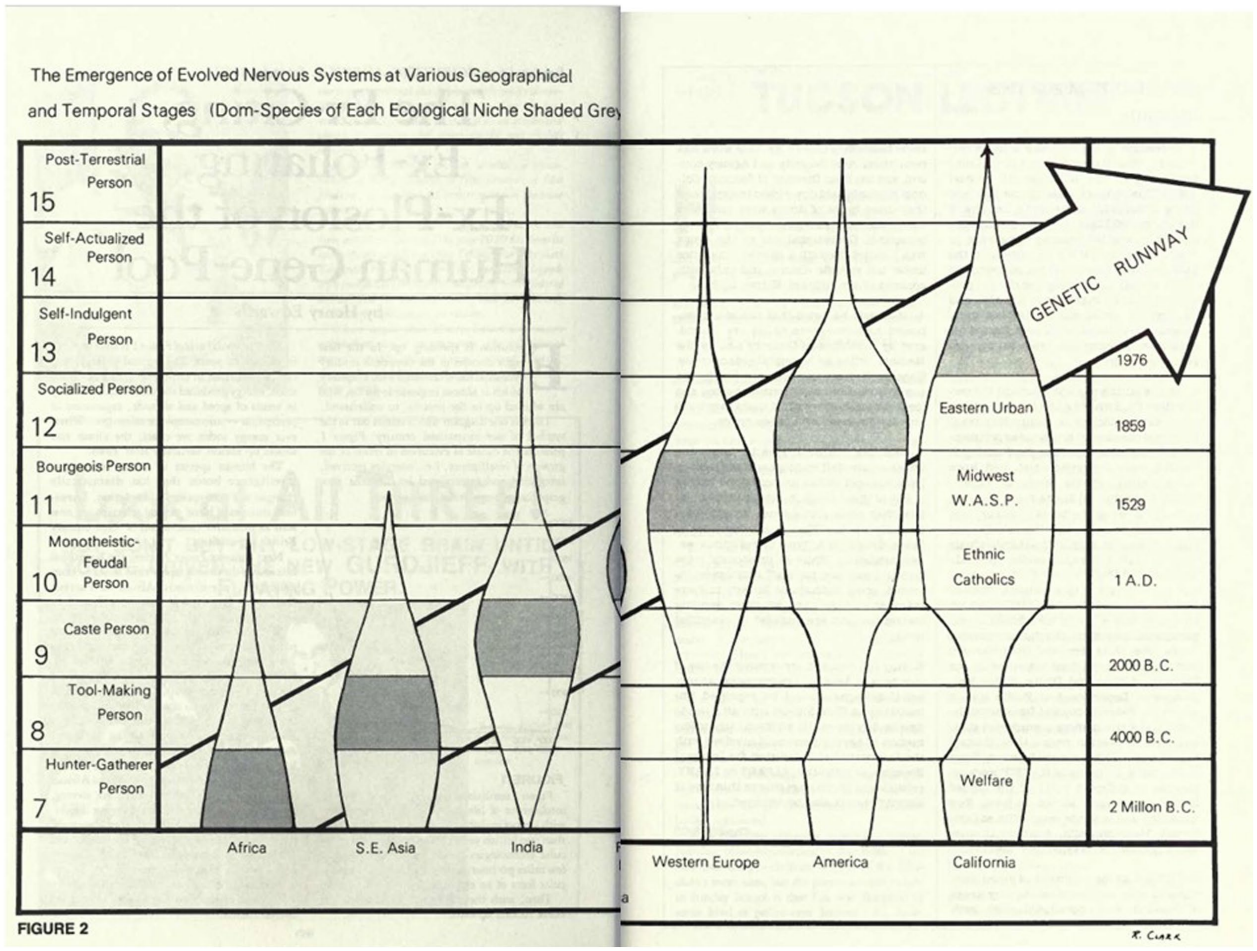
A diagram from Learys’s *Intelligence Agents* (1979). Reproduced with permission from Timothy Leary’s son, Zach Leary, who is responsible for the Leary literary estate.

A graph from Leary’s Intelligence Agents (1979) depicting the varying development of various ‘genetic castes’ (Figure 1).

The most extreme example of evolutionary elitism in the human potential movement is Rajneesh Bhagwan, also known as Osho. He predicted the evolution of ‘the new man’ from within his movement, and told journalists: ‘only the very rich, educated, intelligent, cultured can understand what I am saying’ (Osho, 2022, vol 1: 1). Everyone else is retarded:

Scientifically, the average mental age of a human being is below thirteen … Those who are retarded will criticize you, condemn you. Ignore them. They are already stepping into their graves, soon they will disappear (Osho, 2022, vol 1: 5).

This contempt for the less-evolved masses can feed into Silicon Valley transhumanism, and the dichotomy between the *ubermensch-*like ‘founder’, the tech-genius, the ‘10X engineer’ on the one hand, and the drone-like hordes of ‘non-player characters’ on the other.

### 2.3. Social Darwinism and Malthusianism

This sense of a sharp divide between the evolved elite and degenerate masses can lead to Social Darwinian and Malthusian attitudes: there are too many humans, and there are too many unfit humans. Let nature do its work to select the fittest while letting the unfit die off. This is one way that evolutionary spirituality can be quite different to older religions like Christianity, where those at the bottom of society are seen as having a place in the cosmic scheme of things, and part of serving God involves trying to help the poorest, weakest or least fortunate.

This new attitude can be traced back to the Reverend Thomas Malthus, a founding theologian of evolutionary spirituality, whose *Essay on the Principle of Human Population* in 1798 suggested that nature (created by a Supreme Being) selects the vigorous specimens while getting rid of the listless, malformed and wasteful, thereby creating progressively better beings. Malthus thought welfare for the poor was misguided, as it would merely encourage them to reproduce more, leading to greater misery and vice (Huzel, 1969). His *Essay* was a defining influence on Darwin, and on champions of evolutionary spirituality like the sociologist Herbert Spencer.

Spencer believed that evolution was guided by a spiritual force he called ‘the Unknowable’, and it naturally led to progressively higher forms through the ‘survival of the fittest’ (a phrase he coined). He wrote, “The whole effort of nature is to get rid of [the unfit], to clear the world of them, to make room for better” (Spencer, 1892, p. 205). He suggested that “a finer type of man than has ever hitherto existed” would naturally evolve, as long as governments do not impede natural selection by excessive support for the poor. His evolutionary spirituality was popular with Gilded Age oligarchs like Andrew Carnegie and John D. Rockefeller (Hofstadter, 1944).

Friedrich Nietzsche, arguably a more enduring influence on evolutionary spirituality than Herbert Spencer, also thought the world was over-populated and the weak and ‘unfit’ should be left to die so that higher beings can evolve. He wrote, “Far too many live and far too long they hang on their branches. Would that a storm came to shake all this rot and worm-food from the tree!” (Nietzsche, 2006, p. 54). His social Darwinism influenced early-20th-century spiritual thinkers like Aleister Crowley, whose Book of the Law declares:

We have nothing with the outcast and the unfit: let them die in their misery. For they feel not. Compassion is the vice of kings: stamp down the wretched & the weak: this is the law of the strong: this is our law and the joy of the world.

To which Crowley adds the commentary, “there is a good deal of the Nietzschean standpoint in this … It is the evolutionary and natural view. Of what use is it to perpetuate the misery of Tuberculosis, and such diseases, as we now do? Nature’s way is to weed out the weak” (Crowley, 2004, p. 11).

HG Wells and Julian Huxley, two early prophets of transhumanism, were both socialists who believed in social provisions for the poor. But they were also members of the Malthusian League (Bashford, 2014, p. 1) who believed that ‘overcrowding of the planet’ is “the fundamental evil out of which all the others … arose” (Wells, 1923, p. 1060). They thought over-population leads to demagogic politics, war, pandemics and environmental collapse. Malthusian attitudes could often be found in later prophets of environmental spirituality, like the biologist Paul Erlich, a preacher of ‘conscious evolution’ and author of the doomsday book, *The Population Bomb* (Erlich, 1968), which wrongly predicted that over-breeding in the Third World would lead to mass starvation in the 1970s.

Another member of the Malthusian League was Annie Besant, the Victorian radical who converted to Theosophy in the middle of her life. Like other Theosophists, Besant believed in spiritual evolution, which guides the “survival of the fittest nations and races” and enacts a ‘cleansing process’ for the “unfit ones – the failures” (Blavatsky, 2012, p. 1565). Besant wrote that when she walked through the slums of Britain, she felt “that for those men and women, as they were, degraded, brutal, drunken, profligate … the best mercy that God could show them would be an earthquake that would swallow the whole great city” (Besant, 2020, p. 31).

One encounters an apocalyptic tone in later prophets of evolutionary spirituality as well. Timothy Leary wrote a ‘declaration of evolution’ in 1968, announcing that the ‘new mutants’ must “acquiesce to genetic necessity [and] detach ourselves” from previous generations. It is time, the declaration announced, “for the old mind to die, so that a new one, with expanded sensitivities, could be born” (Leary, 1970). Rajneesh Bhagwan/Osho declared:

I want to be finished with the whole past completely, I want it to be erased completely … Only then the new humanity is possible, a new world, a new man … This world is not worth saving … it will be better if the third world war happens and destroys this whole stupid humanity (Osho, 2022, vol 1: 1).

A good example of this desire for an apocalyptic separation of the fit from the unfit can be found in the thinking of Barbara Marx Hubbard, a champion of evolutionary spirituality and transhumanism from the 1970s to the 2010s. Hubbard claimed her cosmic mission was to midwife the birth of a new species, *homo universalis*. In books like *The Evolutionary Testament of Co-Creation* (Hubbard, 2015), she channeled and interpreted the message of God/Evolution to the ‘evolutionary children of God’ (Hubbard, 2015, p. 126). She described evolution as a mountain — most only get a little way up, but a few special beings get to the top and become intergalactic immortal gods:

At the top we meet everyone who has also kept growing … in a universe full of other beings who have also had the courage to rise to the tops of their mountains, on their own planets in galaxies everywhere … Our time is coming. We who survive the transition … shall identify increasingly with the perspective of God as we build new worlds, create new microorganisms and redesign our bodies for cosmic time and space (Hubbard, 2015, p. 39, p. 74).

Not everyone will make the evolutionary Leap. There will be a ‘bifurcation of humanity’ and selection of the fit from the unfit. It’s up to humans if they make the cut or not:

Individually we can choose to embrace options for evolutionary choices such as longevity, space migration and evolved consciousness. Those who choose these paths will evolve differently from those who choose to remain in the terrestrial/mammalian life cycle … (Hubbard, 2015, p. 59). Just as Neanderthal man passed away, so too will self-centered Homo sapiens retire once it has finished the work of preparing the way for Homo universalis … we will weed out the unworkable from the workable (Hubbard, 2015, p. 96).

As Nietzsche had argued, the elite need to be steely in their acceptance of the passing away of the unfit.

Evolution is compassionate, but not nice … There are missions of mercy to nurse the sick … There is also the new mission of the future: a mission to the strong, the whole, the builders, the scientists, the artists … The New Order of the Future consists of self-selected souls attracted to the future of the world (Hubbard, 2015, p. 36, 106, 369).

It is, of course, offensive for a rich white heiress to suggest those less fortunate than her have somehow ‘chosen’ not to evolve and failed a cosmic evolutionary test. Her prophecy of a bifurcation between the evolved superbeings and the left-behind masses finds echoes in contemporary transhumanism.^5^ Psychedelic entrepreneur Christian Angermayer, who founded atai Life Sciences, has suggested:

humanity could split up into two species, because you have a part of humanity who says ‘hey bring it on, lets fly to Mars’. If you want to go to Mars you will need to change your bodies. We’re going to have to modify, everyone knows it. But there might be a part of humanity who says ‘this is not for me, I don’t want to merge with machines’. So humanity might split into two species (The Rubin Report, 2021).^6^

One notes Social Darwinian attitudes in some psychedelic transhumanists, particularly in their rejection of mass welfare democracy in favour of offshore crypto-libertarianism. The Extropian movement of the 1990s, who included some of the founders of cryptocurrency, were typically anarcho-libertarians who believed in exiting the nation-state and starting up their own crypto utopias, where they could engineer themselves into superbeings, free from the intrusion of the IRS and FDA (Evans, 2022c). That dream lives on in contemporary transhumanists like psychedelic and crypto investor Peter Thiel, who funded the Seasteading Institute to explore the possibility of crypto-libertarian offshore utopias, in expectation of the collapse of mass welfare democracies (Lanier and Glenn Weyl, 2022).^7^

### 2.4. Spiritual eugenics

The sense of a coming bifurcation between the Elect and the Passed Over is not unique to evolutionary spirituality. But in Abrahamic religions, it is God who selects the Wheat from the Chaff. In evolutionary spirituality, in some instances at least, it is humans who select the fit and the unfit. As Nietzsche put it, “the ruling caste of the future … must now take the place of God … They deliver the physiologically botched by teaching them the doctrine of ‘swift death’”(Nietzsche, 1911, p. 266).

It is not the case that all the leading figures in pre-war evolutionary spirituality supported eugenics in one form or another. Many did not, and a few actively opposed it, like William James and Alfred Russell Wallace. But it’s worth noting how many leading figures *did* support eugenics in some form: Friedrich Nietzsche, George Bernard Shaw, Ernst Haeckel, Julian and Aldous Huxley, HG Wells, Gerald Heard, WB Yeats and several other members of the Golden Dawn, Rudolf Steiner and other German Theosophists, Jan Smuts, several members of the Society for Psychical Research, Sri Aurobindo, Luther Burbank, John Harvey Kellogg, and Alexis Carrel all supported positive and/or negative eugenics to steer evolution and produce higher beings. One also occasionally finds a support for eugenics in post-war evolutionary spirituality, for example in the thinking of Abraham Maslow, Aldous and Julian Huxley, Teilhard de Chardin, William Luther Pierce and Osho.^8^

Eugenics was a science-religion launched by Francis Galton, Charles Darwin’s cousin, in 1883. Galton noted that many human characteristics, including intelligence and some illnesses and disabilities, seemed to be hereditary. He suggested that the science of heredity could be combined with animal breeding techniques to steer evolution and create ‘master-minds’ (Galton, 1865). Although eugenics has typically been described as a pseudo-science, it was embraced by its followers as an evolutionary religion (Galton, 1909, p. 42), which would replace *homo sapiens* with a ‘new born Apollo’ (Pearson, 1930, p. 217). Scientists would be the priests of this new religion, measuring and quantifying the value of humans through psychometric and biometric tests to sort the superior from the inferior. These scientist-priests would guide human evolution by encouraging the ‘fittest’ to breed more and with each other (positive eugenics) and discouraging those deemed ‘unfit’ from breeding at all (negative eugenics) through voluntary or involuntary sterilization, segregation, racial miscegenation laws and, at its most extreme, euthanasia, or mass murder (Bashford and Levine, 2010). This ‘jehad or Holy War’ (Galton, 1909, p. 99) would save civilization from being swamped by imbeciles (Huxley, 1994, p. 150).

The eugenic ‘religion of the future’ was extremely popular from the 1880s to the 1940s, across the political spectrum and in many countries of the world. Many different policies and activities were promoted in eugenic terms as helping to create a superior race or species, including everything from body-building and yoga to organic farming and psychedelics. For example, in 1941, the German poet-soldier-physician Gottfried Benn called for a “systematic educational effort in the direction of conscious enhancement of vitality. One could, by increasing visionary states say with mescaline or hashish, supply the race with a stream of spiritual insights which could lead to a new creative period” (Benn, 2007, p. 44). The fact that psychedelics could be promoted using eugenic rhetoric does not mean one can say psychedelic culture ‘is’ eugenicist (or body-building, organic farming or yoga). Rather, it is indicative of the general popularity of eugenic rhetoric before World War Two.

But quite often, those who used eugenic rhetoric to promote a particular policy or activity also supported positive and/or negative eugenics. Gottfried Benn, for example, wrote in his 1933 essay ‘Eugenics’, written following the Nazis’ ascent to power, “It seems to me certain that once again a new man will emerge from this transformation in Europe, half as a mutation and half as a result of eugenics” (Benn, 2013, p. 207). Several other important figures in western psychedelic history saw both psychedelics and eugenic breeding laws as technologies to assist the evolution of superbeings, and this overlap highlights an illiberal tendency in evolutionary spirituality.

For example, the first British paper on psychedelics was published by Havelock Ellis in 1897. Ellis was a free-thinking, left-leaning physician, one of the first English disciples of Nietzsche, and a follower of evolutionary spirituality. He believed in the possibility of steering evolution to develop ‘the divine possibilities of man’ (Ellis, 1912, p. 404), and he suggested that mescaline was one method to enhance humans’ evolutionary potentialities (Ellis, 1898). He also believed some humans are degenerating, and that the ‘feeble-minded’ ‘dilute the spiritual quality of the community’. Ellis was a member of the Eugenics Education Society from 1907 to 1939 and supported the voluntary sterilization of those deemed ‘unfit’. Eugenic policies would bring about a new millennium, he believed, “Not until the earth is purified of untold millions of its population will it ever become the heaven of old dreamers, in which the elect walk spaciously and nobly, loving one another” (Ellis, 1912, p. 404).

One of the participants in Ellis’ mescaline experiments was his friend WB Yeats, the Irish poet and member of the Hermetic Order of the Golden Dawn (Jay, 2019, p. 92). Yeats, like other adepts, used occult techniques in his attempt to ascend up the spiritual-evolutionary scale, including mind-altering drugs such as hashish and mescaline. At the other end of the evolutionary scale, Yeats suggested that western civilization was in danger of collapse because “the better stocks have not been replacing their numbers, while the stupider and less healthy have been more than replacing theirs.” He thought state-enforced eugenics was necessary, “Sooner or later we must limit the families of the unintelligent classes” (Yeats, 1962, p. 423).

HG Wells and Julian Huxley, two early transhumanists, promoted both mind-altering drugs and eugenics as technologies to guide human evolution towards the creation of superbeings. In *The Science of Life*, a 1929 book co-written by HG Wells, his son Gip Wells and Julian Huxley, the authors note the recent discovery of psychoactive drugs like mescaline, and remark, “It is not only that these drugs illuminate our capabilities. A time will come when they may be used to assist and enhance them” (Wells et al., 1934, p. 1388). They also say the new science of eugenics is essential for counteracting a steep rise in “defectives,” “pockets of evil germ-plasm responsible for a large amount of vice, disease, defect and pauperism” (Wells et al., 1934, p. 1470). In his 1901 book *Anticipations,* Wells suggested a biological underclass might need to be exterminated (Wells, 1902, p. 300), although he later moved to the slightly-more-liberal position of Julian Huxley, who suggested the ‘unfit’ could be bribed into getting sterilized (Weindling, 2012).

Julian’s brother, Aldous, promoted eugenic policies from the 1920s until his death in 1962, as a means of lowering the quantity and raising the quality of the population. His 1931 novel, *Brave New World*, is frequently read an anti-eugenic dystopian satire. However, his essays from the late 1920s and early 1930s show the extent to which he supported the sort of caste-based scientific-eugenic dictatorship proposed in *Brave New World.*^9^ In a radio broadcast in 1932, Aldous said, “in a scientific civilization society must be organized on a caste basis. The rulers and their advisory experts will be a kind of Brahmins controlling, in virtue of a special and mysterious knowledge, vast hordes of the intellectual equivalent of Sudras and Untouchables.” He called for “a society compelled by law to breed more and more exclusively from its most gifted and socially most successful members.” (Huxley, 1994, pp. 113, 152).

In the last decade of his life, Aldous embraced an optimistic evolutionary spirituality influenced by his brother’s transhumanism. In 1961, he declared:

I think there are still a great many potentialities … still lying latent in man. And it may be that … we will find methods for going beyond where we are now, in a few hundred years, as far as we have succeeded in going beyond our Aurignacian ancestors in 20,000 years (Huxley, 1961).

He thought psychedelics were one method to expand human potentialities – his friend, the psychiatrist Humphrey Osmond, suggested psychedelics would enable a new form of human to evolve. And Aldous still endorsed eugenics as another method for expanding human potential. In his 1958 lecture series at UC Santa Barbara, he said:

Sooner or later eugenics will be practiced, although it is certainly going to take a tremendous revolution in our present ethical ideas on the subject. It may be added that the first nation that does practice such eugenic methods will in a few decades be enormously superior to all its rivals (Huxley, 1978, p. 105).

Abraham Maslow also believed humans could evolve through ‘peak experiences’, attained by psychedelics or other means. And he privately expressed support for negative eugenics and even euthanasia to raise the genetic quality of the species. In his journals one can find comments such as:

We keep alive many of the people whom nature left to itself would kill off. So we are hurting the human gene pool, which must be deteriorating … The right to reproduce must surely become rather a privilege which is socially controlled and socially granted … (Maslow, 1979, p. 833) … One day we’ll have to talk about the exposure or killing of monster-babies, or even of healthy surplus babies (Maslow, 1979, pp. 1230–1231).

In a paper on ‘Humanistic Biology: Elitist Implications of the Concept of Full Humanness’, which he delivered at the Salk Institute in 1968, he suggested the ‘biological aristocracy’ should become ‘a kind of priestly class’, which decides who gets to reproduce and who does not:

Who is to judge how to evolve ourselves, which type of individual should be favored and selected, or who is to live and to die?..Will the decision makers be a federal commission, a global board, or a special group of physicians, biologists, or other scientists?..The question of how to select the most adequate and wise, the best people to make these awe-full decisions must, therefore, be considered an urgent program (Maslow, 1996a,b).

Another leading figure in the human potential movement, Osho, argued that, to create the ‘new man’, there needs to be a global eugenic law:

We need more stronger bodies, we need more intelligent people, and we need people who are clean of all old crap. That is possible only if we make a clinical, medical arrangement for the birth of man … bioengineering certainly can create far superior men, healthier, more talented (Osho, 2022, 1: 3).

He said there should be a total global ban on births for 20 years, and then a medical-spiritual board of control should regulate all reproduction, authorizing births through artificial insemination so that only genetically-gifted children were born. The human population should be reduced by 75%. Aid to the third world “should completely stop.” (Osho, 2022, 1: 5).

From the 1970s on, transhumanists have tended to propose voluntary genetic enhancement, rather than coercive eugenic breeding laws, as a means to steer evolution and create superbeings. Figures like Timothy Leary in the 1970s, or Nick Bostrom and David Pearce in the 1990s, argued for humans’ right to genetic enhancement (see Evans, 2022b,c). Leary suggested that a genetic ‘elite of elites’, 5,000 humans selected for their genetic superiority, should jet into space, establish an off-world colony, and there use genetic technologies to create a more intelligent, blissful and longer-living species (Leary, 1974). This may be far-fetched and elitist, but is not an illiberal as 1920s-style enforced mass eugenics.^10^ Instead, transhumanists defend what Nicholas Agar has called ‘liberal eugenics’ (Agar, 1998) – your right to modify yourself and your children, free from government interference.

But sometimes transhumanists still argue for state-sponsored programmes of biochemical and genetic enhancement, and these do seem potentially illiberal and coercive. For example, www.eugenics.org is a website launched by British philosopher David Pearce, who co-founded the World Transhumanist Association in 1994. He suggests ‘a bio-happiness revolution is imminent’ and wants to create a more blissful ecosystem, using techniques ranging from psychedelics to ‘wireheading’ to genetic engineering. He says, “Genome reform to engineer lifelong loved-up MDMA-like consciousness would be my vision of paradise” (What’s It Like To Be A Philosopher, 2022). He looks forward to the day when the World Health Organization can alter our genes and raise our hedonic level with one injection. His plan to ‘abolish suffering’ entails the genetic modification not just of humans but of all species. He insists this is eugenics, but ‘not 1920s-style eugenics’ (Evans, 2022a). His intentions are of course altruistic, nonetheless, here the line between ‘liberal eugenics’ and enforced eugenics seems perilously thin.

### 2.5. Illiberal medical-spiritual utopias

The biggest ethical problem with evolutionary spirituality, as I see it, is its marriage of science and religion. Every religion has its particular values and prejudices, but apostles of evolutionary spirituality insist their dogmas are objective ‘empirical facts’, as Aldous Huxley liked to say. They invariably commit what GE Moore called the naturalistic fallacy—they shift from the Is of scientific data to the Ought of moral preaching. And often the data is extremely weak. With eugenics, psychiatrists could deem someone a ‘moral defective’ based on a snap judgement, confining them to incarceration, sterilization or even extermination if, for example, they had a child outside of marriage, or were the wrong ethnicity, or even if they smiled too much. This was William James’ critique of eugenics. He wrote, “The trouble is that [eugenicists] use the descriptive names of symptoms merely as an artifice for giving objective authority to their personal dislikes. The medical terms become mere ‘appreciative’ clubs to knock men down with” (Richardson, 2006, p. 338). Eugenics is obviously an extreme case but I see a similar risk in any science-religion that claims it can quantify people according to their level of self-actualization as more or less ‘fully human’.

If the dogma of science-religions become enshrined in laws, you have a risk of what Alfred Russell Wallace (referring to eugenics) called a ‘medical tyranny’ run by ‘an arrogant scientific priestcraft’ (Wallace, 2003, p. 214). All religions are potentially illiberal when imposed onto an entire population, but science-religions are insidiously so, because their devotees insist they are not imposing their particular theology onto a populace, but rather an ‘objective science’ of flourishing/self-actualization/mystical oneness (Evans, 2018). It’s worth noting that many of the leading champions of evolutionary spirituality argued for illiberal and anti-democratic utopian projects. HG Wells, Julian and Aldous Huxley, Gerald Heard, Alexis Carrell, Sri Aurobindo, Abraham Maslow and Osho all put forward political schemes in which a Platonic caste of scientist-priests decide how the rest of us should think, live and breed. In all of their spiritual utopias, there is one presumed goal for the entire population – the evolution of humanity to a higher state. All means and all lives serve that goal and those who do not agree with it are excised from the body-politic. Multicultural, liberal, secular society is replaced by a medical-mystical cult.

Aldous Huxley’s *Island,* for example, is often celebrated as a hippy utopia, and it inspired Esalen and later spiritual communes. Huxley describes Pala as a decentralized democracy which encourages non-dogmatic critical thinking. Yet it is a completely closed, static, theocratic society, in which there is one presumed goal for all: mystical self-actualization. There is one neuro-theological culture, established by the Raja and an enlightened Scottish doctor a century before and unchanged since. Every citizen of Pala is indoctrinated in this culture, through the island’s bible, mantras, hypnotherapy and the psychedelic ritual that every child must go through to become an adult. Population is strictly controlled, and the nuclear family is replaced by collective ‘mutual adoption clubs’. Second children are encouraged to be born *via* artificial insemination from genius sperm banks, to raise the general IQ of the society. Any delinquents are spotted as infants and given drugs for the rest of their life. Huxley suggests this will be about one fifth of all males. And if they are not cured? “In the long-run … they always are” (Huxley, 2005, p. 154).

His friend Gerald Heard, another important influence on psychedelic culture in the 1950s and 1960s, preached a similar medical-spiritual utopia in his 1939 book, *Pain, Sex and Time*. Heard argued that humanity had reached the limit of its current evolutionary phase, and needs to advance to the next level and become superbeings. This required intense spiritual training. Heard sees two possibilities. Either the mass of humanity will degenerate and go extinct, while a few special humans gather in spiritual ‘Collegiums’ and evolve to the next stage; or a new type of human arises, which he calls ‘Neo-Brahmins’, and through sheer charisma they lead humanity into a ‘new order’. This goes beyond liberal democracy, which Heard says is ruled by lower appetites, and is instead a medical-spiritual caste-based theocracy with one central goal, “the further evolution of consciousness beyond individuality.” He writes:

Instead of a remnant being saved, a few pioneers getting through to the new level, the whole vast column of mankind … might be in its entirely shifted upwards toward an increasing awareness … of the comprehensive purpose of their existence (Heard, 1939, p. 310).

This new order would be non-violent, he insists, though he also says all of humanity would be guided by an ‘International Police Force’, a cross between policemen, psychiatrists and priests, dedicated to helping humanity achieve ‘complete liberation from the self’. Again, this is a rejection of secular, liberal, multicultural society in favor of a medical-mystical theocratic cult.^11^

## 3. Conclusion

What I have described are tendencies in the tradition of evolutionary spirituality, and for each of these tendencies, one can find exceptions and counter-examples. A good counter-example to the tendency to spiritual narcissism and spiritual eugenics is William James, who denounced eugenics perhaps because he recognized his own mental vulnerability, and also because he was a pluralist. He thought human consciousness could evolve in many different directions, some of which might seem aberrant or even pathological to outsiders. You cannot fit all of humanity into one map of development – there are many potential peaks in the ‘fitness landscape’ and not all of them can necessarily be quantified and measured scientifically. I suspect James would be appalled that psychedelic science now seeks to grade people’s mystical experiences on a scale from 1 to 10 (Evans, 2018).

In response to evolutionary spirituality’s tendency to social Darwinism and contempt for the unevolved masses, one could re-incorporate traditional religious virtues and beliefs, such as a belief in the essential value of human life, and a commitment to humility, charity and service to others, particularly those less fortunate than you. As for evolutionary spirituality’s tendency to illiberal utopian projects, a good counterexample would be John Stuart Mill. He believed in the possibility of self-cultivation to higher states of being, but did not think you should impose one model of the good life onto an entire society, least of all a pseudo-scientific religion like Comte’s ‘religion of humanity’, which Mill accused of ‘spiritual despotism’ (Mill, 1873, p. 213).

Instead of imposing one model of self-actualization onto humanity, Mill argued for a secular, liberal, tolerant framework within which multiple ‘experiments in living’ could be pursued. An example of this sort of Millsian ‘experiment in living’ might be Esalen, an organisation dedicated to evolutionary spirituality, which has avoided the cultishness of other human potential movements thanks to two principles: ‘hold your dogmas lightly’ and ‘no one captures the flag’ (Kripal, 2007). Of course, Esalen has been accused of being a country club, only accessible to the well-off. How could one make such programmes pluralist, non-coercive, as safe as possible, and accessible to those without great wealth, but with an inclination to follow a particular form of training? ^12^

As to ‘spiritual eugenics’, the risk of coercive 1920s-style eugenic programs seems low today. But we do see transhumanists and biotech entrepreneurs (including some prominent investors in psychedelics like Peter Thiel and Christian Angermayer) arguing for individuals’ right to alter their genes as well as their consciousness. This ‘liberal eugenics’ raises a different ethical dilemma – not the risk of the violent imposition of genetic technologies onto the masses, but the risk of new genetic technologies being only available to the wealthy. Already, we are seeing an underground market for genetic enhancement technologies like embryo selection by polygenic risk scores, which are only available to the wealthy and well-connected (Black, 2022). We’re seeing the rise of ‘genetic tourism’ like ‘psychedelic tourism’—the rich go to Costa Rica for ayahuasca retreats, and Cyprus for stem-cell injections. The fact that genetic enhancement technologies are largely confined to the wealthy has led some to express concern that humanity could bifurcate into two species—GenRich (the genetically enriched) and Naturals (Silver, 1997). This concern seems hyperbolic, but certainly health, education and income inequalities could get a lot worse.

Today, transhumanism—the leading contemporary form of evolutionary spirituality—is effectively a religion for the extremely rich of Silicon Valley. Billionaires like Elon Musk, Sergey Brin, Larry Page, Mark Zuckerberg, Peter Thiel, Christian Angermayer, Steve Jurvetson, Larry Ellison and others believe in humanity’s capacity to evolve into superhumans through technologies like AI, VR, genetic modification and psychedelics (Evans, 2022c). They see a glorious intergalactic future, but not necessarily for everyone, not in the short-term anyway. There is a risk the ultra-rich could retreat into offshore and off-world gated ashrams to enhance themselves and weather out the apocalypse while everyone else suffers decades of climate change and system collapse (Rushkoff, 2022).

If transhumanism remains merely a religion for the rich and powerful, it is unlikely to survive. Already it has provoked an anti-transhumanist backlash—the conspiracy-obsessed masses rail against the invidious agenda of the globalist elite to turn themselves into superbeings while culling the rest of us (Istvan, 2014). The more such anti-transhumanist conspiracy theories flourish, the more there is a risk the general public will reject new technologies and the progress of science will be slowed. We need to communicate the benefits of new technologies (including psychedelics and genetic medicines), and make them affordable, safe, and accessible. This would be democratic transhumanism (Hughes, 2004). The more it focuses on healing ordinary people of sickness, rather than creating an elite of superbeings, the more popular it will be.

But why turn evolution into a religion at all? Why worship new technologies or the coming superbeings? Aldous Huxley said humans weave religions like spiders weave webs (Huxley, 2005, p. 177). We cannot help it. And maybe religions play a useful role in inspiring people and giving them a sense of meaning and purpose. But religions are also prone to dogmatism, apocalyptic eschatologies and collective spiritual narcissism. Evolutionary spirituality is no different. And it’s not necessarily more rational, evidence-based or true than other religions.

Thomas Huxley, the great Victorian scientist and grandfather of Aldous and Julian, started off promoting the religion of science, and suggested evolution could teach us moral values. But by the end of his life, he became more agnostic (a word he coined) and decided evolution was not a good basis for ethics, religion or politics. In ‘Evolution and Ethics’ (Huxley, 1997, pp. 283–304) he points out that what is evolutionarily fitter is not necessarily what is morally better. And the church of evolution often leads to arrogant scientist-priests ranking human beings in value and even dictating who deserves to live and breed. Is it necessary, desirable or scientifically-valid to fasten one’s spirituality onto evolutionary theories? I do not think so. It’s possible to believe in spiritual development without thinking it somehow makes you ‘more evolved’.

## Data availability statement

The original contributions presented in the study are included in the article/Supplementary material, further inquiries can be directed to the corresponding author.

## Author contributions

The author confirms being the sole contributor of this work and has approved it for publication.

## Funding

This research was supported by the Wellcome Trust’s Living with Feeling grant to the Centre for the History of the Emotions at Queen Mary, University of London.

## Conflict of interest

The author declares that the research was conducted in the absence of any commercial or financial relationships that could be construed as a potential conflict of interest.

## Publisher’s note

All claims expressed in this article are solely those of the authors and do not necessarily represent those of their affiliated organizations, or those of the publisher, the editors and the reviewers. Any product that may be evaluated in this article, or claim that may be made by its manufacturer, is not guaranteed or endorsed by the publisher.

## Footnotes

^1^I pondered whether to include Deep Ecology and ‘Gaia religions’ in this list. What Bron Taylor calls ‘Dark Green Religion’ (Taylor, 2010) is certainly a form of post-Darwinian evolutionary religion, and it can share some of the tendencies I highlight in this essay. What it does not so often share with other forms of evolutionary spirituality is a dream of humans evolving into superhumans. Some forms of ‘Gaia religion’ are quite antithetical to transhumanism, even though both can be traced back to pioneers like Julian Huxley. On the other hand, Gaia prophet James Lovelock’s final book embraces the transhumanist idea of the Singularity (Lovelock, 2019).

^2^Hofmann told David Jay Brown, "I do not know if [LSD] has affected evolution, but I hope so" (Brown, 2015). Aldous and Julian Huxley both believed that humans are evolving new potentialities, including through the use of mind-altering drugs (Huxley, 1933, p. 68; Huxley, 1961). Humphrey Osmond, in correspondence with Aldous Huxley, suggested psychedelics could help *homo sapiens* evolve into *‘homo faber’* (Huxley and Osmond, 2018, p. 230). Timothy Leary thought LSD was interacting with human DNA and consciousness to create a ‘new race of mutants’ (The Harvard Crimson, 1966), and his Harvard colleague Ralph Metzner explored this idea in his essay ‘On the Evolutionary Significance of Psychedelics’ (Metzner, 1968). Robert Anton Wilson, in his book *Prometheus Rising,* argued that humans could evolve into ‘cosmic immortals’ *via* occult methods including psychedelics (Wilson, 1983). Terence McKenna suggested psychedelics play central role in human evolution in his 1992 book *Food of the Gods* (McKenna, 1992). Stan Grof discusses consciousness evolution in Grof (1996). Rick Doblin has said that psychedelics could create a ‘core of evolved humanity’ (Doblin, 2014).

^3^I do not suggest all forms of evolutionary spirituality are inherently racist, far from it. Julian Huxley, for example, is one of the most important thinkers in the tradition of evolutionary spirituality, and he strenuously opposed scientific racism from the 1930s until his death in 1977. There are also forms of evolutionary spirituality that see racial interbreeding as aiding the evolution of mixed-race superbeings (eg Jose Vasconcelos’ *Cosmic Race*) although this can lead to its own forms of racism. One extremely racist version of evolutionary spirituality is Cosmotheism, a white supremacist eugenic religion launched in the 1970s by William Luther Pierce, who is more famous as the author of the far-right novel, *The Turner Diaries* (Whitsel, 1998).

^4^Theosophists protest that, according to their theory of spiritual evolution, all souls reincarnate through various races. Nonetheless, some racial incarnations are seen as higher, purer and more evolved than others.

^5^In *LSD and the Mind of the Universe* (Bache, 2019), philosopher Chris Bache recounts taking high doses of LSD over several years and communicating with the Mind of the Universe. It tells him there will be a ‘Great Death’ and ‘global purification’ of the species followed by the evolution of the ‘future human’.

^6^In fact, Angermayer thinks psychedelics could be an ‘antidote for future shock’, helping the alienated to cheer up and join the Singularity.

^7^This sort of transhumanist libertarianism may not be indicative of everyone in the tradition of evolutionary spirituality of course. In Pew Research’s 2014 study of the ‘Spiritual But Not Religious’ in the US, a majority believe government aid to the poor does more good than harm, though a near-majority also believed in smaller government: https://www.pewresearch.org/religion/religious-landscape-study/religious-denomination/spiritual-but-not-religious/

^8^On Nietzsche’s support for eugenics, see Holub, 2018, pp. 408–454. On George Bernard Shaw and WB Yeats’ support for eugenics, see Carey, 1992, pp. 13–22. Other members of the Golden Dawn and affiliated organisations supported positive and/or eugenics, including Isabelle de Steiger, Florence Farr, Aleister Crowley and Dion Fortune, see Evans (2021c). For Haeckel’s comments in support of euthanasia and infanticide, see Weikart (2002). On Julian Huxley’s support for positive and negative eugenics, see Weindling, 2012. On Aldous Huxley’s support for positive and negative eugenics, see Huxley, 1994, pp. 105–114, 147–158. On HG Wells’ support for negative eugenics, see Partington (2002). Gerald Heard promoted eugenics in talks and articles like ‘Eugenics and the New Order’ (Heard, 1933) – see also Fallby (2020), p. 33. For Steiner’s views on racial evolution and miscegenation, see Staudenmaier (2014). Jan Smuts supported laws on segregation and miscegenation as prime minister of South Africa (Schwarz, 2011, pp. 277–340). For the overlap between membership of the Eugenics Society and the Society for Psychical Research see Evans (2022d). On Sri Aurobindo’s call for eugenic breeding laws, see Singleton, 2007. For Luther Burbank’s support for positive eugenics, see Burbank, 1907. For the place of eugenics in Kellogg’s ‘religion of biologic living’, see Wilson, 2014, pp. 148–168. On Alexis Carrel’s fusion of eugenics and evolutionary religion, see Reggiani, 2006. For Teilhard de Chardin’s comments in support of eugenics, see Slattery, 2017. For Luther Pierce’s racist eugenic religion, see Whitsel, 1998. For Maslow and Osho’s comments on eugenics, see quotes later in the essay.

^9^"Our civilization is menaced with total collapse. Dictatorship and scientific propaganda may provide the only means for saving humanity from the misery of anarchy." (Huxley, 1994, p. 111).

^10^Leary, ever the provocateur, did say Hitler was an evolutionary visionary for his support of eugenics (see Leary and Wilson, 1978, p. 254).

^11^Could such utopias work as small voluntary spiritual communes, rather than as prescriptions for entire societies? Maybe, but even at the level of the commune, evolutionary spirituality seems often to lead to authoritarian and illiberal cults, like those of Osho, Andrew Cohen, Scientology or NXIVM. In seeking authenticity and liberation, devotees can end up enslaved to the whims of their charismatic leader, mindlessly repeating slogans. But this may emerge from cult dynamics rather than anything specific to evolutionary spirituality.

^12^It’s worth noting that the first time Aldous Huxley discussed places for the development of human potentialities, it was after vising an unemployment centre during the Great Depression. He argued that such places could become ‘universities for common life’ where people are able to explore and develop their preferred potentialities, for free (Huxley, 1994, p. 235).
